# Supplementary material for: Ebola and Marburg virus matrix layers are locally ordered assemblies of VP40 dimers
Source: eLife. 2020 Oct 5;9:e59225. doi: 10.7554/eLife.59225 (PMC7588233; doi:10.7554/eLife.59225)
Supplement: Transparent reporting form [file elife-59225-transrepform.docx]

***eLife’s* transparent reporting form**

We encourage authors to provide detailed information *within their submission* to facilitate the interpretation and replication of experiments. Authors can upload supporting documentation to indicate the use of appropriate reporting guidelines for health-related research (see [EQUATOR Network](http://www.equator-network.org/%20)), life science research (see the [BioSharing Information Resource](https://biosharing.org/)), or the [ARRIVE guidelines](http://www.plosbiology.org/article/info:doi/10.1371/journal.pbio.1000412) for reporting work involving animal research. Where applicable, authors should refer to any relevant reporting standards documents in this form.

If you have any questions, please consult our Journal Policies and/or contact us: [editorial@elifesciences.org](mailto:editorial@elifesciences.org).

**Sample-size estimation**

- You should state whether an appropriate sample size was computed when the study was being designed
- You should state the statistical method of sample size computation and any required assumptions
- If no explicit power analysis was used, you should describe how you decided what sample (replicate) size (number) to use

Please outline where this information can be found within the submission (e.g., sections or figure legends), or explain why this information doesn’t apply to your submission:

For electron microscopy, sample sizes were determined by available electron microscopy time and density of particles on electron microscopy grids. Dataset sizes are shown in Table S2. The numbers of observations and unique reflections for crystallographic data are added in Table S1.

**Replicates**

- You should report how often each experiment was performed
- You should include a definition of biological versus technical replication
- The data obtained should be provided and sufficient information should be provided to indicate the number of independent biological and/or technical replicates
- If you encountered any outliers, you should describe how these were handled
- Criteria for exclusion/inclusion of data should be clearly stated
- High-throughput sequence data should be uploaded before submission, with a private link for reviewers provided (these are available from both GEO and ArrayExpress)

Please outline where this information can be found within the submission (e.g., sections or figure legends), or explain why this information doesn’t apply to your submission:

For our submission, biological replicates are defined as sample preparation while technical replicates would be each independent observation, i.e. each tilt-series (which may contain one or more VLPs/virions). Biological replicates were generally performed once for this study, but reproduce the same morphological features described by us and others in the literature. Technical replicates are listed in Table S2. All acquired tilt-series were analyzed and data was later excluded as is usual for such image processing studies. First, tilt-series that showed poor fiducial alignments or imaging problems were removed from the dataset. Similar to single-particle EM reconstruction, poorly aligned subtomograms were excluded from averages based on cross-correlation scores and visual analysis of lattice maps.

The experiments in Figure S4 were repeated three times as described in the figure legend.

**Statistical reporting**

- Statistical analysis methods should be described and justified
- Raw data should be presented in figures whenever informative to do so (typically when N per group is less than 10)
- For each experiment, you should identify the statistical tests used, exact values of N, definitions of center, methods of multiple test correction, and dispersion and precision measures (e.g., mean, median, SD, SEM, confidence intervals; and, for the major substantive results, a measure of effect size (e.g., Pearson's r, Cohen's d)
- Report exact p-values wherever possible alongside the summary statistics and 95% confidence intervals. These should be reported for all key questions and not only when the p-value is less than 0.05.

Please outline where this information can be found within the submission (e.g., sections or figure legends), or explain why this information doesn’t apply to your submission:

Our submission describes a number of determined structures, and as such statistics concerning hypothesis testing do not apply. Statistics for the crystal structures are described in Table S1. Resolution estimates for the subtomogram averaging structures used a ‘gold-standard’ approach of independently refined half-maps for Fourier Shell Correlation calculations. Statistical tests in Figure S4 are described in the legend to that figure.

(For large datasets, or papers with a very large number of statistical tests, you may upload a single table file with tests, Ns, etc., with reference to sections in the manuscript.)

**Group allocation**

- Indicate how samples were allocated into experimental groups (in the case of clinical studies, please specify allocation to treatment method); if randomization was used, please also state if restricted randomization was applied
- Indicate if masking was used during group allocation, data collection and/or data analysis

Please outline where this information can be found within the submission (e.g., sections or figure legends), or explain why this information doesn’t apply to your submission:

For independent subtomogram alignment, datasets were split into odd and even subsets based on subtomogram number. Subtomogram numbers are effectively randomized per virion or virus-like particle. This is described in the materials and methods section.

**Additional data files (“source data”)**

- We encourage you to upload relevant additional data files, such as numerical data that are represented as a graph in a figure, or as a summary table
- Where provided, these should be in the most useful format, and they can be uploaded as “Source data” files linked to a main figure or table
- Include model definition files including the full list of parameters used
- Include code used for data analysis (e.g., R, MatLab)
- Avoid stating that data files are “available upon request”

Please indicate the figures or tables for which source data files have been provided:

The source data for the graph in Figure S2d is here:
